# Supplementary material for: Mitochondrial CISD1/Cisd accumulation blocks mitophagy and genetic or pharmacological inhibition rescues neurodegenerative phenotypes in Pink1/parkin models
Source: Mol Neurodegener. 2024 Jan 25;19:12. doi: 10.1186/s13024-024-00701-3 (PMC10811860; doi:10.1186/s13024-024-00701-3)
Supplement: Supplementary file 1 — Additional file 1: Supplementary Figure S1. Characterisation of Cisd in ageing and different tissues. (A) Immunoblot analysis of protein lysates from young (2 days) or old (30 days) whole flies of the indicated genotypes. Samples were homogenised under non-reducing (top panel) and reducing (bottom panel) conditions. Blots were probed for Cisd (CISD2, Proteintech, 13318-1-AP) and Tubulin. (B) Immunoblot of mitochondrial fractions from WT flies, probed for poly-Ub, Cisd (CISD2, Proteintech, 13318-1-AP) and ATP5a. (C) Immunoblot analysis of protein lysates from 2- and 30-day-old adult heads of the indicated genotypes, probed for pUb, Cisd (CISD2, Proteintech, 13318-1-AP) and Tubulin. (D) Relative amount of Cisd monomer and dimer quantified from replicate blots shown in C. (E) Immunoblot of protein lysates from whole flies of WT or Cisd null (−/−) or Cisd overexpression (OE) driven by da-GAL4, probed for Cisd (CISD2, Proteintech, 13318-1-AP) and Tubulin. (F, G) Climbing assay of 2- and 20-day-old WT flies orCisd overexpression driven only in (F) DA neurons (TH-GAL4) or (G) pan-muscle (Mef2-GAL4). (H) Lifespan analysis of flies overexpressing Cisd in muscles as in G versus a WT control genotype. Statistical analysis: (D) unpaired t-test; (F, G) Kruskal-Wallis non-parametric test with Dunn’s post-hoc correction. ** P < 0.01, **** P < 0.0001. Supplementary Figure S2. Quantification of p62, Atg8-II and Cisd protein levels, and Pink1 and parkin mRNA. (A, B) Quantification of immunoblots shown in Fig. 4D. (C) Quantification of Cisd levels from immunoblots shown in Fig. 4I. (D, E) Quantification of Pink1 (D) or parkin (E) transcript levels upon Cisd overexpression (OE) or knockdown (RNAi). Statistical analysis: (A, B, D, E) Welch’s t test, (C) one-way ANOVA with Sidak’s post-hoc correction. * P < 0.05, ** P < 0.01, **** P < 0.0001. Supplementary Figure S3. Characterisation of Cisd loss-of-function on organismal and mitochondrial phenotypes. (A) Immunoblot analysis of protein l [file 13024_2024_701_MOESM1_ESM.pdf]

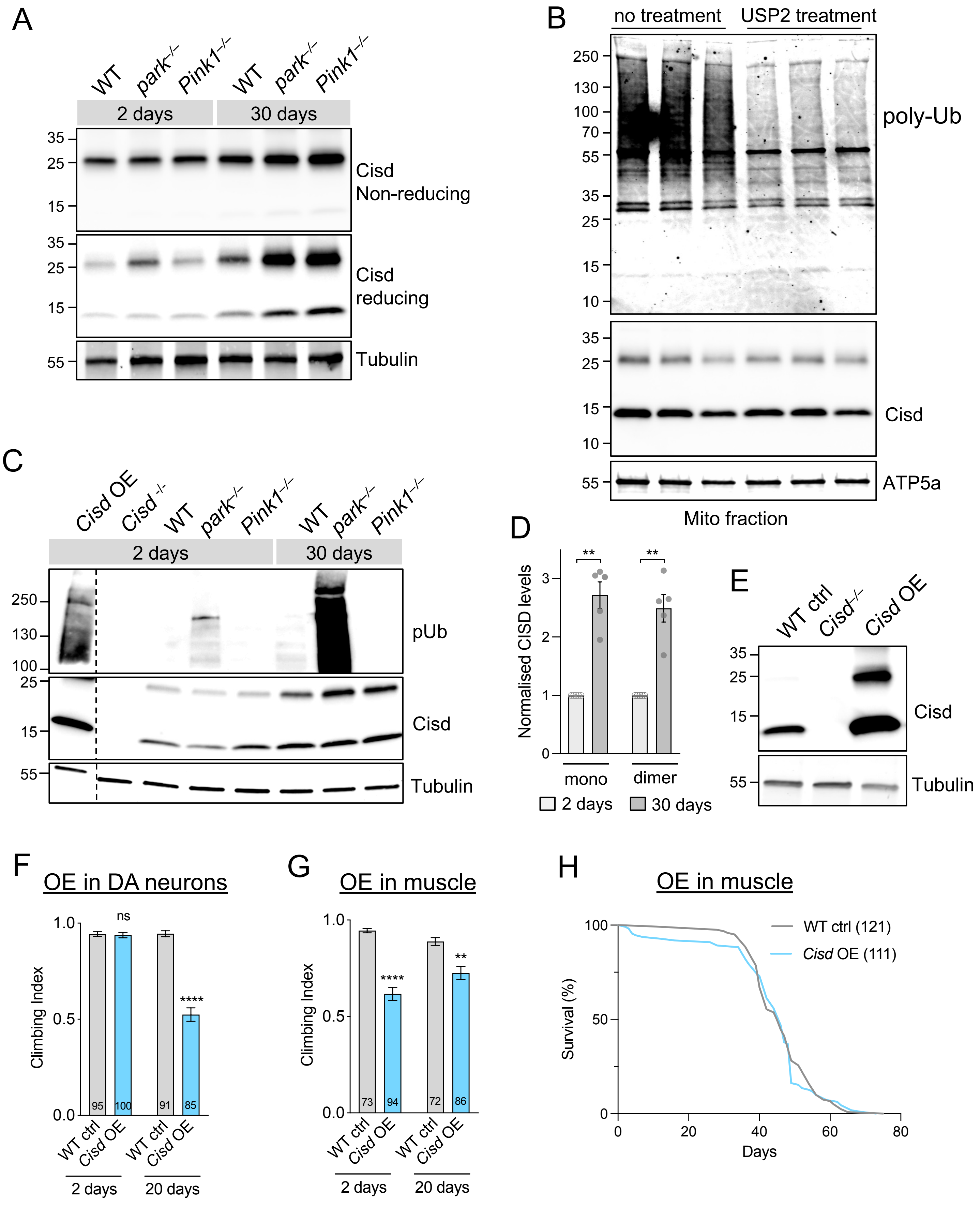

A
WT
park-/-
Pink1-/-
2 days
30 days
Cisd Non-reducing
Cisd reducing
Tubulin
B
no treatment
USP2 treatment
poly-Ub
Cisd
ATP5a
Mito fraction
C
Cisd OE
Cisd -/-
pUb
D
Normalised CISD levels
mono
dimer
2 days
30 days
E
WT ctrl
Cisd-/-
Cisd OE
F
OE in DA neurons
Climbing Index
ns
20 days
G
OE in muscle
H
OE in muscle
WT ctrl (121)
Cisd OE (111)
Survival (%)
Days

Figure S1

Supplementary Figure S1. Characterisation of *Cisd* in ageing and different tissues

(A) Immunoblot analysis of protein lysates from young (2 days) or old (30 days) whole flies of the indicated genotypes. Samples were homogenised under non-reducing (top panel) and reducing (bottom panel) conditions. Blots were probed for Cisd (CISD2, Proteintech, 13318-1-AP) and Tubulin. (B) Immunoblot of mitochondrial fractions from WT flies, probed for poly-Ub, Cisd (CISD2, Proteintech, 13318-1-AP) and ATP5a. (C) Immunoblot analysis of protein lysates from 2- and 30-day-old adult heads of the indicated genotypes, probed for pUb, Cisd (CISD2, Proteintech, 13318-1-AP) and Tubulin. (D) Relative amount of Cisd monomer and dimer quantified from replicate blots shown in C. (E) Immunoblot of protein lysates from whole flies of WT or *Cisd* null (−/−) or *Cisd* overexpression (OE) driven by *da*-GAL4, probed for Cisd (CISD2, Proteintech, 13318-1-AP) and Tubulin. (F, G) Climbing assay of 2- and 20-day-old WT flies or *Cisd* overexpression driven only in (F) DA neurons (*TH*-GAL4) or (G) pan-muscle (*Mef2*-GAL4). (H) Lifespan analysis of flies overexpressing *Cisd* in muscles as in G versus a WT control genotype. Statistical analysis: (D) unpaired t-test; (F, G) Kruskal-Wallis non-parametric test with Dunn's post-hoc correction. ** $P < 0.01$, **** $P < 0.0001$.

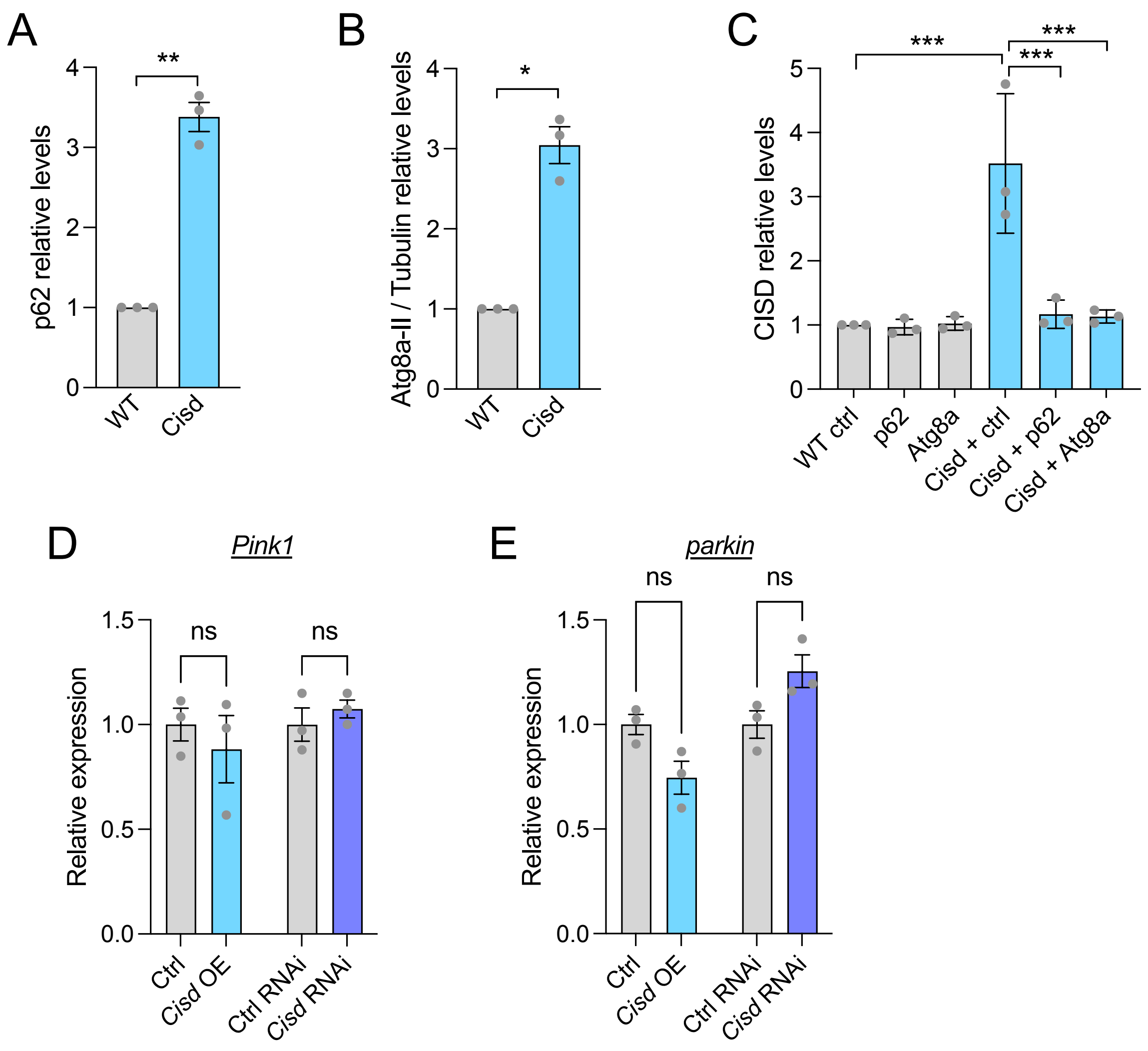

A
**
p62 relative levels
0
1
2
3
4
WT
Cisd
B
*
Atg8a-II / Tubulin relative levels
0
1
2
3
4
WT
Cisd
C
***
***
***
CISD relative levels
0
1
2
3
4
5
WT ctrl
p62
Atg8a
Cisd + ctrl
Cisd + p62
Cisd + Atg8a
D
Pink1
ns
ns
Relative expression
0.0
0.5
1.0
1.5
Ctrl
Cisd OE
Ctrl RNAi
Cisd RNAi
E
parkin
ns
ns
Relative expression
0.0
0.5
1.0
1.5
Ctrl
Cisd OE
Ctrl RNAi
Cisd RNAi

Figure S2

Supplementary Figure S2. Quantification of p62, Atg8-II and Cisd protein levels, and *Pink1* and *parkin* mRNA.

(A, B) Quantification of immunoblots shown in Fig. 4D. (C) Quantification of Cisd levels from immunoblots shown in Fig. 4I. (D, E) Quantification of *Pink1* (D) or *parkin* (E) transcript levels upon *Cisd* overexpression (OE) or knockdown (RNAi). Statistical analysis: (A, B, D, E) Welch's *t* test, (C) one-way ANOVA with Sidak's post-hoc correction. * $P < 0.05$, ** $P < 0.01$, **** $P < 0.0001$.

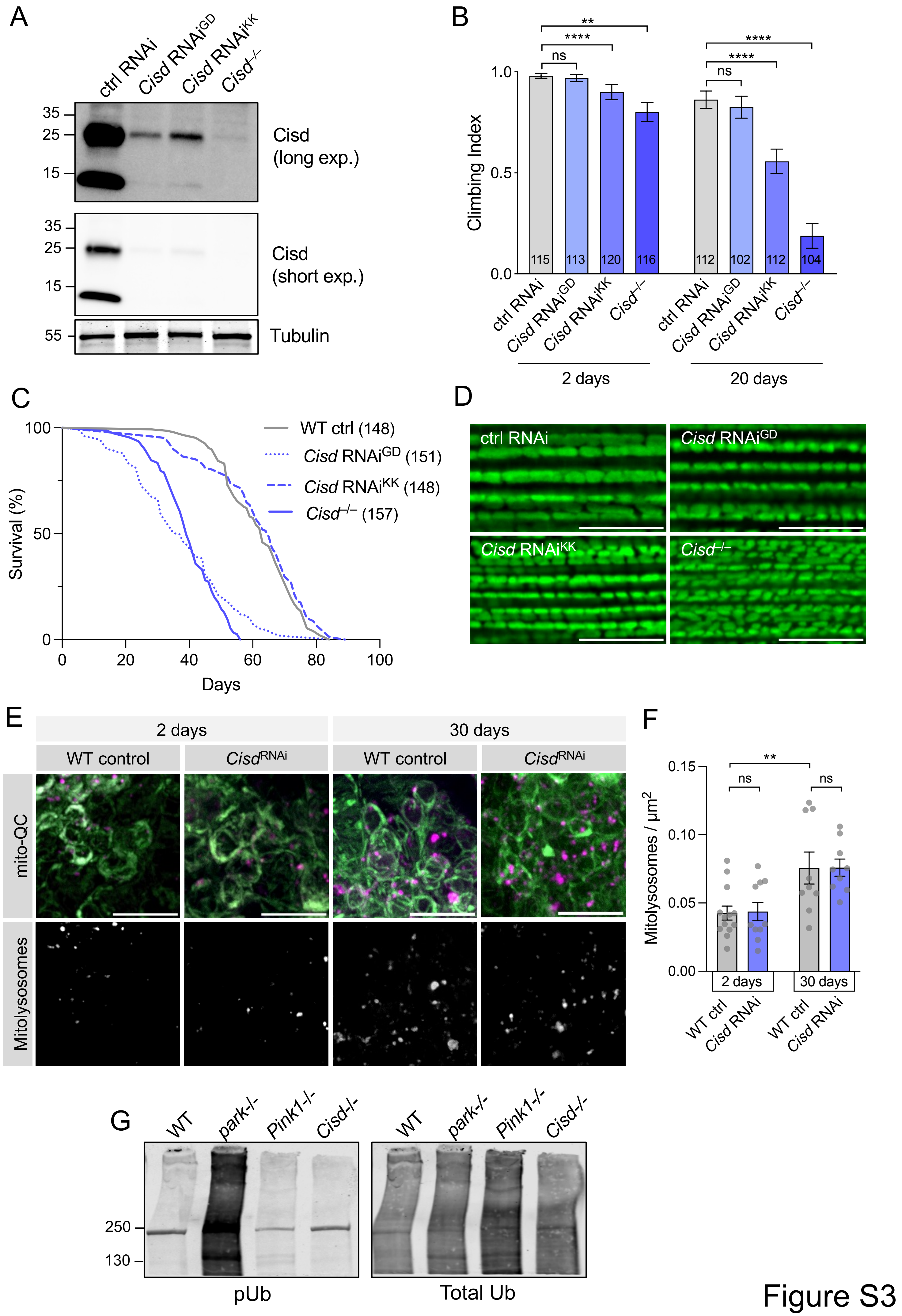
A
ctrl RNAi
Cisd RNAi^GD
Cisd RNAi^KK
Cisd−/−
35
25
15
Cisd
(long exp.)
Cisd
(short exp.)
55
Tubulin
B
Climbing Index
**
****
ns
****
ns
****
115
113
120
116
112
102
112
104
2 days
20 days
C
Survival (%)
Days
WT ctrl (148)
Cisd RNAi^GD (151)
Cisd RNAi^KK (148)
Cisd−/− (157)
D
ctrl RNAi
Cisd RNAi^GD
Cisd RNAi^KK
Cisd−/−
E
2 days
30 days
WT control
Cisd^RNAi
mito-QC
Mitolysosomes
F
Mitolysosomes / μm²
**
ns
2 days
30 days
WT ctrl
Cisd RNAi
G
WT
park-/-
Pink1-/-
Cisd-/-
250
130
pUb
Total Ub

Figure S3

Supplementary Figure S3. Characterisation of *Cisd* loss-of-function on organismal and mitochondrial phenotypes

(A) Immunoblot analysis of protein lysates from whole flies of the indicated genotypes of *Cisd* loss versus control, probed for Cisd (CISD2, Proteintech, 13318-1-AP) and Tubulin. (B) Climbing assay of 2- and 20-day-old flies of the indicated genotypes. Knockdown is driven by *da*-GAL4. (C) Lifespan analysis of *Cisd* loss as in B. (D) Confocal microscopy analysis of mitochondrial morphology, immunostained for ATP5A mitochondrial marker in flight muscle of the indicated genotypes. Knockdowns are driven by *da*-GAL4. (E) Confocal analysis of adult neurons of the indicated ages, WT control and *Cisd* RNAi (KK line) animals co-expressing the mitophagy reporter *mito*-QC (OMM-localised tandem RFP-GFP) with *nSyb*-GAL4 to highlight mitolysosomes, shown separately and quantified in F. Statistical analysis: (B) Kruskal-Wallis non-parametric test with Dunn's post-hoc correction. (F) one-way ANOVA with Sidak's post-hoc correction. * $P < 0.05$, **** $P < 0.0001$. Scale bars = 10 µm. (G) Immunoblot analysis of protein lysates from whole flies of the indicated genotypes probed for pUb and Total Ub.

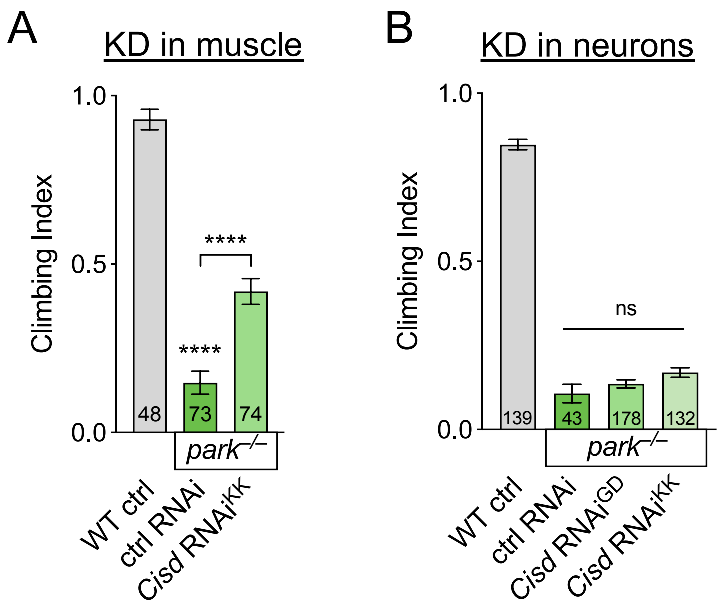

C

	**ctrl OE**	***Cisd* OE**
**WT**	viable	viable
***park*$^{-/-}$**	viable	lethal
***Pink1*$^{-/-}$**	viable	lethal

Figure S4

Supplementary Figure S4. Characterisation of *Cisd* and *parkin* genetic interaction

(A, B) Climbing assay of tissue-specific *Cisd* knockdown (KD) in either pan-muscles with *Mef2*-GAL driver (A) or pan-neurons with *nSyb*-GAL driver (B) alongside respective controls. (C) Viability assay for genetic interactions between the indicated combinations revealing synthetic lethality of *Pink1/parkin* mutants and *Cisd* OE with *da*-GAL driver. (D) Confocal microscopy analysis of mitophagy reporter *mito*-QC in flight muscle from *Cisd* knockdown driven by *Mef2*-GAL4 in WT and *parkin* mutant backgrounds in 30-day-old animals. (E) Quantification of the number of mitolysosomes shown in D. Data points indicated individual animals analysed. Statistical analysis: one-way ANOVA with Sidak's post-hoc correction; * $P < 0.05$, **** $P < 0.0001$. Scale bars = 10 µm.
